# An Approach to Overcome the Limitations of Surveillance of Asbestos Related Diseases in Low- and Middle-Income Countries: What We Learned from the Sibaté Study in Colombia

**DOI:** 10.5334/aogh.4166

**Published:** 2023-10-04

**Authors:** Juan Pablo Ramos-Bonilla, Margarita Giraldo, Daniela Marsili, Roberto Pasetto, Benedetto Terracini, Agata Mazzeo, Corrado Magnani, Pietro Comba, Benjamin Lysaniuk, María Fernanda Cely-García, Valeria Ascoli

**Affiliations:** 1 Universidad de los Andes, Bogotá, Colombia; 2 Collegium Ramazzini, Bologna, IT; 3 Departamento de Ingeniería Civil y Ambiental, Universidad de Los Andes, Bogotá, Colombia; 4 Department of Environment and Health, Istituto Superiore di Sanità, ISS, Rome, IT; 5 WHO Collaborating Centre for Environmental Health in Contaminated Sites, Istituto Superiore di Sanità, Rome, IT; 6 Unit of Cancer Epidemiology, University of Torino and CPO-Piemonte, Torino, IT; 7 Department of History and Cultures, University of Bologna, Bologna, IT; 8 Department of Translational Medicine, University of Eastern Piedmont, Novara, IT; 9 IRD (MàD by CNRS), UMR Prodig, Aubervilliers, FR; 10 Department of Radiological, Oncological and Anatomo-Pathological Sciences, Sapienza University, Rome, IT

**Keywords:** Asbestos, Sibaté, Colombia, Mesothelioma, Cluster, Surveillance, Research-protocol, Asbestos-cement-facility, non-occupational-exposure

## Abstract

**Introduction:**

The asbestos industry began its operations in Colombia in 1942 with the establishment of an asbestos-cement facility in Sibaté, located in the Department of Cundinamarca. Despite extensive asbestos use and production in Colombia, the country lacks a reliable epidemiological surveillance system to monitor the health effects of asbestos exposure. The Colombian health information system, known as SISPRO, did not report mesothelioma cases diagnosed in the municipality, posing a significant challenge in understanding the health impacts of asbestos exposure on the population of Sibaté.

**Methods:**

To address this issue, an active surveillance strategy was implemented in Sibaté. This strategy involved conducting door-to-door health and socioeconomic structured interviews to identify Asbestos-Related Diseases (ARDs). Validation strategies included a thorough review of medical records by a panel of physicians, and the findings were communicated to local, regional, and national authorities, as well as the general population.

**Results:**

The active surveillance strategy successfully identified a mesothelioma cluster in Sibaté, revealing the inadequacy of the existing health information system in monitoring asbestos-related diseases. The discovery of this cluster underscores the critical importance of implementing active surveillance strategies in Colombia, where governmental institutions and resources are often limited.

**Conclusion:**

The findings of this study emphasize the urgent need for Colombia to establish a reliable epidemiological surveillance system for asbestos-related diseases (ARDs). Active surveillance strategies can play a crucial role in identifying mesothelioma clusters and enhancing our understanding of the health effects of asbestos exposure in low- and middle-income countries.

## 1. Introduction

Asbestos, in all its forms, is universally recognized as a carcinogen to humans, causing a spectrum of diseases that includes mesothelioma, lung cancer, laryngeal cancer, ovarian cancer, asbestosis, and non-neoplastic pleural lesions [1]. By December 2022, asbestos had been banned in 69 countries [2], including six Latin American countries, Colombia being one of the countries that recently approved a complete asbestos ban in 2019 [3]. However, despite these bans, asbestos remains legally used in many low- and middle-income countries, which frequently grapple with inadequate health and environmental institutions to effectively address this persistent hazard.

The asbestos industry started operations in Colombia in 1942 when an asbestos-cement facility was established in Sibaté, a city located 25 Km from the capital, Bogotá [4]. Subsequently, additional asbestos cement facilities emerged in Cali and Barranquilla (1944), along with two in Manizales in 1967 and 1982 [4]. Additionally, an asbestos friction products plant commenced operations in Bogotá in 1960, while a chrysotile asbestos mine began operations in 1980 in the municipality of Campamento (Antioquia) [4]. Unfortunately, the available information concerning these facilities remains quite limited, and the current and cumulative number of workers involved in these operations is unknown.

In response to concerns raised by local inhabitants, the Sibaté study was initiated in early 2015. Over time, an international consortium of researchers from Colombia, Italy, and France has contributed to a series of publications that have unveiled several critical findings: 1) [5,6,7] the first mesothelioma cluster that has been reported in Colombia; 2) landfilled zones with the presence in specific locations of an underground layer of friable asbestos (see Table 1); 3) mesothelioma patients diagnosed at a very young age, suggesting exposure during childhood or adolescence; and 4) the occurrence of non-occupational exposures in the city, since only one out of 13 certain mesothelioma cases studied in detail worked in the asbestos-cement facility.

**Table 1:** Summary of the findings of the soil sampling campaigns conducted in Sibaté [5,7].


LOCATION	NUMBER OF SAMPLES POSITIVE FOR ASBESTOS	DEPTH OF THE ASBESTOS CONTAINING LAYER (CM)	DESCRIPTION	ASBESTOS TYPE CHRYSOTILE (CHRY) CROCIDOLITE (CROC)	ASBESTOS CONTENT (%)

Outside Stadium	6	10–110	Friable asbestos	Chry	5–10%

Public school	1	35	Friable asbestos	Chry/Croc	10% Chry 2% Croc

Soccer field	9	44–80	Friable and non friable asbestos, soil	Chry/Croc	5–10% Chry 2% Croc


When reviewing studies that report a relatively high incidence rate or mortality from mesothelioma in various regions or countries worldwide, common characteristics emerge. Firstly, the majority of these studies documenting an excess number of cases are conducted in high-income countries. Secondly, most of these studies rely on a combination of global, national, and regional vital statistics and health information systems, often incorporating cancer, and, in some cases, mesothelioma registries. In our analysis, we identified studies reporting a relatively high incidence rate or mortality from mesothelioma using cancer registries in Italy (i.e., National Bureau of Statistics – ISS [8]; National Mortality Database – ISS [9], Italian National Institute for Statistics [10,11], case control study of Casale Monferrato [12], cohort study of Casale Monferrato) [13], Belgium (i.e., WHO Mortality Database (WMD)) [14], The Netherlands (i.e., Dutch Cancer Register) [15], United Kingdom (i.e., Thames Cancer Registry) [16], Germany (i.e., German Federal Statistical Office and WMD) [17], Spain (i.e., National Statistics Institute [18], Spanish Employment Ministry) [19], Denmark (i.e., Danish Cancer Registry) [20], Brazil (i.e., DATASUS Database) [21], United States (i.e., Libby-based Center for Asbestos Related Diseases [22], Public Health Service Hospital in Santa Fe, New Mexico [23], Connecticut Tumor Registry [24], California Cancer Registry [25], New Jersey State Cancer Registry) [26], China (i.e., Da-yao county hospital records [27] and Yuyao People’s Hospital Patients) [28], Peru (i.e., Mortality data from Peru Ministry of Health – MINSA) [29], South Korea (i.e. Korea Central Cancer Registry) [30], and France (Cancer Registry of New Caledonia) [31,32]. There are also studies about the incidence of mesothelioma in multiple European countries [33], using the WHO/International Agency for Research on Cancer (IARC) Database.

Moreover, several studies employ mesothelioma registries to determine the frequency and distribution of mesothelioma cases in specific countries and regions. Examples include Italy [34,35,36,37,38,39,40,41], the United Kingdom (i.e., British Mesothelioma Register) [42], and Australia (i.e., Western Australia Mesothelioma Registry) [43,44,45,46,47]. It is crucial to acknowledge the limited number of mesothelioma registries worldwide. A literature review identified mesothelioma registries in Italy, France, the United Kingdom, Australia, and South Korea [48]. A study reported additional mesothelioma registries in Belgium, Germany, Japan, South Africa, and Turkey [49]. Another article reported mesothelioma registries in different Latin American countries (i.e., Argentina, Brazil, Colombia, Costa Rica, Panamá, México, Peru, Nicaragua, and Venezuela), pooled in a database called MeSO-CLICaP Platform [50]. The MeSO-CLICap database represents a one-time initiative by the Latin American Consortium for Lung Cancer Research (CLICAP), led by a team of dedicated oncology researchers. This database was established to retrospectively document and report on cases of mesothelioma identified within their clinical practice [51]. However, this database is not publicly available [51].

Similar to the protocols applied in the Sibaté study, some studies conducted in Turkey identified mesothelioma cases directly in the field by interviewing the patients or their families, and assessed potential asbestos exposure [52,53,54].

This paper outlines the methods employed during the Sibaté study, which aimed to investigate a substantial number of asbestos related diseases (ARD) cases reported by the inhabitants of the municipality, in the absence of reliable health databases usable for assessing the occurrence of any ARD or a mesothelioma registry. The aim of this paper is to provide a practical guide to investigators engaged in bringing to light previously undetected, if not undetectable, localized excesses of ARD whose discovery might open the way to environmental cleanup, health protection, and increased awareness of asbestos burden of diseases in the affected communities.

## 2. Materials and Methods

The information available in the Colombian official morbidity and mortality database (SISPRO) was not reliable, since it did not identify the ARD cases that local inhabitants were denouncing [5]. Consequently, a different approach was used to investigate the public health situation in Sibaté. Based on this experience, we describe a protocol that can be followed to determine if there is an excess number of ARD in a region where an asbestos-processing facility or a mine currently operates or operated in the past, or where there is a suspicious of an unusual occurrence of ARD without knowledge of any sources of asbestos exposure.

**Figure 1:**
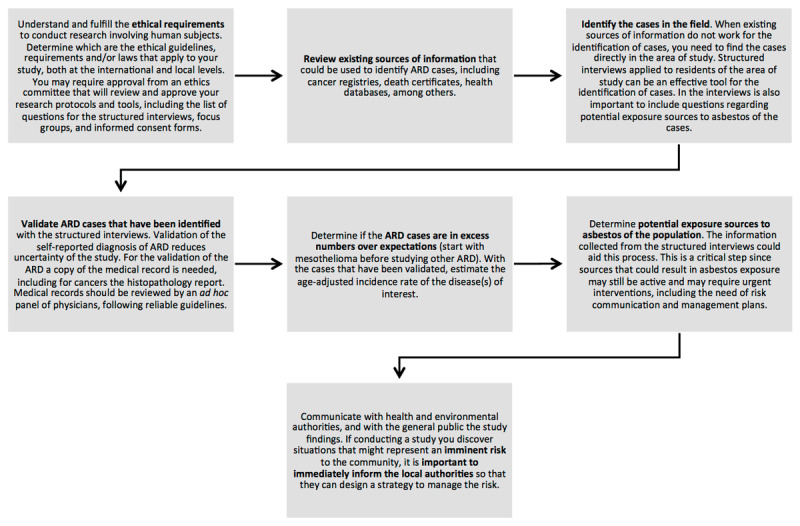
Protocol to determine if there is an excess number of ARD in a region.

The proposed protocol includes the following steps, and is shown in Figure 1:

1. **Understand and fulfill the ethical requirements:** Begin by comprehending and adhering to the ethical requirements for conducting research involving human subjects. Determine which are the ethical guidelines, requirements, and laws that apply to your study at both international and local levels. You may need approval from an ethics committee, responsible for reviewing and approving your research protocols and tools, including interviews, focus groups, and informed consent forms. In fact, such approval is often a requirement of scientific journals for publication. This step should precede all other project activities. If your institution lacks an Institutional Review Board (IRB) or equivalent (e.g., an ethics committee) to approve your project, you may need to seek approval from another committee for your study. Several international ethical guidelines can be consulted for this step, including the Belmont Report [55] and the Guiding Principles for Ethical Research of the U.S. National Institutes of Health [56].
2. **Review existing sources of information:** Examine existing sources of information that could be utilized to identify cases of Asbestos-Related Diseases (ARD), such as cancer registries, death certificates, and health databases, among others. Ensure the obtained information is valid and reliable. Does the morbidity and mortality data align with what you are observing in the field? Do the numbers make sense? Ensure compliance with ethical requirements identified in Step 1, as assessing some of these sources may necessitate prior ethical approval.
3. **Identify the cases in the field:** When existing sources of information are insufficient for identifying cases, you must directly locate cases within the study area. If feasible, engage in participative communication to coordinate this stage with local authorities. Additionally, try to identify community leaders who can assist in this phase. Identifying cases entails an intensive field search, often conducted through face-to-face structured interviews with members of the target population. Several considerations for this stage include:
  - **Ethical Approval for Research Tools:** Note that the research tools you employ, both qualitative and quantitative, may require ethical approval.
  - **Informed Consent:** Prior to participating in the study, volunteers must have read, comprehend, and signed informed consent forms.
  - **Design of Interview Questions:** Carefully design the questions for the interviews, possibly involving key social actors with a direct interest in the topic. Test the proposed interview model with focus groups as part of the research tool development process, especially considering the potential limited literacy on asbestos risk and ARD among participants. Testing the structured interview and assembling focus groups also necessitates ethical approval and informed consent. While designing interview questions, verify that you gather all the necessary information for the study’s reliability. Provide study participants with contact information through which they can reach the research team if they have questions or wish to provide additional information. Furthermore, inquire whether study participants would be available for future contact by the research team, either for clarification of their responses or for subsequent study phases. For epidemiological estimates required later in the investigation of ARD, the minimum information needed from each patient includes: i – Gender, ii – Age, iii – Current residence, iv – Previous residences, v – Age at the time of disease diagnosis, vi – Diagnostic center, vii – Years of residence at the study location, viii – Identity of the person answering the structured interview (patient or family member), ix – Occupational history, x – Age of death (if applicable), xi – History of potential asbestos exposure events. Additionally, demographic information from other participants that have not been diagnosed with an ARD should also be collected.
  - **Monitor Non-Respondents:** Keep a record of the number of non-respondents of the structured interview so that you can calculate the response percentage.
  - **Establish Eligibility Criteria:** Define the criteria for eligibility for inclusion in the structured interview and, if necessary, for obtaining a representative sample of eligible persons.
  - **Geographical Information:** It is crucial to collect the location information of respondents in the structured interview. If possible, supplement their place of residence with geographical coordinates.
  - **Identify Asbestos Exposure Sources:** Ideally during the structured interview, you should attempt to identify potential sources of asbestos exposure for the cases. This includes both occupational and residential history, hobbies and activities that may involve interactions with asbestos, and presence of asbestos containing materials in current and previous residences or in other facilities where the patient spent time.
4. **Validate ARD cases:** Validate ARD cases identified through structured interviews. When an individual (or a family member) reports an ARD diagnosis, it requires validation. Validation is crucial because there can be confusion in the naming of diseases. For example, in the Sibaté study, we discovered that many people use the term “asbestosis” for diseases associated with asbestos exposure, but the original diagnosis might be mesothelioma or lung cancer. Furthermore, mesothelioma is a rare and challenging disease to diagnose. Therefore, validating self-reported ARD diagnosis reduces study uncertainty. To validate ARD cases, a copy of the medical record, including histopathology report for cancers, is needed. Even if the medical record confirms the reported ARD, it is not sufficient for proper validation. The medical record should be reviewed by an ad hoc panel of physicians following reliable guidelines.
5. **Determine Excess ARD cases:** Determine if the number of ARD cases exceeds expectations. For validated cases, estimate the age-adjusted incidence rate of the disease(s) of interest [57,58]. If previous research has not been conducted, we recommend focusing on mesothelioma before studying other ARDs, since mesothelioma is specifically associated with asbestos and is considered a sentinel disease of asbestos exposure [59]. To assess whether there is an excess number of cases, we recommend estimating the age-adjusted incidence rate, since it allows for a straightforward comparison with rates observed in other regions and countries worldwide. For ARD information, a valuable source is the IARC report *Cancer Incidence in Five Continents*, which is periodically updated. The latest report (i.e., XI) to date was released on March 2021 [60].
6. **Determine Asbestos Exposure Sources:** Determine potential sources of asbestos exposure in the population. The information collected from the structured interview could assist in this process. This is a critical step because sources leading to asbestos exposure may still be active and may require urgent interventions, including risk communication and management plans. Since the highest asbestos exposures tend to occur in occupational settings, it is important to determine the gender distribution of the workforce at the asbestos-processing facility in the study area. Analyze whether the incidence rates by gender provide additional insights into the situation. If the workforce is predominantly male and you observe high incidence rates in females, you may have important exposure sources outside the asbestos-processing facility, including para-occupational exposures. To standardize exposure classification, the categories proposed in the Italian Mesothelioma National Register [61] are a useful guideline.
7. **Communicating Findings:** Communicate the study findings with health and environmental authorities and the general public [62,63,64]. If during the study you discover situations that might represent an imminent risk to the community, promptly inform local authorities so they can design a risk management strategy. When communicating findings to the public, consider several aspects: 1 – do not to generate panic, 2 – respect individuals and protect the anonymity of patients and their families, 3 – if the media are involved, provide scientific information in plain language to ensure the delivery of accurate messages. Writing a media statement can be very helpful. It is good practice to wait for peer validation of results before disseminating study findings to the public. This reduces the risk of disseminating incorrect or misleading results that could misinform and harm the public. The World Health Organization offers free online courses on risk communication on emergency situations [65].

## 3. Results

The protocol described above was applied in the Sibaté study in the following way:

### 1. Fulfilling Ethical Requirements to Conduct Research Involving Human Subjects

In the case of the Sibaté study, which involved structured interviews, focus groups, and the collection and review of patients’ medical records, approval was required from the ethics committee of Universidad de los Andes, the research institution that initiated the study. The ethics committee at Universidad de los Andes was established and operates in compliance with both Colombian legal requirements (Resolution 8430/1993) [66], and international ethical guidelines. The committee approved the list of questions for the structured interviews, the informed consent form, and the conduct of focus groups to validate the structured interviews. During the course of the study, additional requests for amendments to the original approval were made, including the collection of medical records [5]. It is essential to note that, in accordance with ethical guidelines, approval must be obtained before conducting any specific research activity.

### 2. Reviewing Existing Sources of Information that Could be Used to Identify ARD Cases

The Sibaté study was initiated in January 2015 in response to reports from residents of the municipality, which were widely covered in mass media, suggesting a high number of ARD cases diagnosed in the area [5]. To address these concerns, the first step was to consult Colombia’s official morbidity and mortality database, known as SISPRO [67]. However, when using the year of diagnosis as a search criterion, no cases were found reported in SISPRO records for the municipality of Sibaté [5]. In SISPRO, reported cases are anonymous, making it impossible to determine whether the cases identified in Sibaté had been reported in another municipality. The explanation provided for this inconsistency was that the information in SISPRO is uploaded by the treating physician, and it is possible that, instead of using the address of the case’s residence, the address of the healthcare facility where the diagnosis was made was used. Based on this, the decision was made to identify cases within the municipality using a structured interview.

### 3. Finding the Cases Directly in Sibaté

Health and socio-economic structured interviews were conducted by researchers of Universidad de los Andes in Sibaté, specifically in four neighborhoods (San Martín, El Carmen, San Rafael, El Progreso) that inhabitants perceived could have asbestos contamination, based on reports from the population that some areas of the municipality were used to dispose asbestos contaminated products [5,6,7] These structured interviews involved the use of paper questionnaires, and were carried out through door-to-door visits, covering all the residences in the four neighborhoods mentioned earlier [5]. The majority of these structured interviews took place between August 2015 and June 2016, although some were conducted later in the study. Most of the interviews were conducted by students enrolled in the Environmental Engineering program at Universidad de los Andes. These students underwent training on how to interact with study participants, and the initial interviews were supervised by senior researchers, with a focus not only on the methods but also on adherence the ethical guidelines.

A total of 355 structured interviews were completed by Sibaté residents, and included questions about the patient or interviewed patient, knowledge about asbestos, the patient’s occupational history, household and family details, potential past asbestos exposure, and health and medical information, including cigarette consumption [5]. One oversight during this process was the failure to track the number of non-responses, which included residents who declined to participate or dwellings were no response was obtained. Given that this was a door-to-door structured interview conducted in four neighborhoods, it was possible to count the total number of dwellings present in these four neighborhoods, enabling the estimation of the response rate, which stood at 23% (270 structured interviews out of a total of 1164 dwellings) [5]. The additional 85 interviews that completed the total of 355 structured interviews were obtained from other areas of the municipality [5].

Responses from the questionnaires were transferred to a spreadsheet for subsequent analysis. Binary codes (dummy variables) were used for multiple choice questions, while the original responses, both numerical and text, were retained for open-ended questions such as age, place of birth, and occupation, among others. In cases where questionnaire responses raised doubts, the interviewer who conducted the interview was contacted for clarification. Information collected from the structured interviews enabled the identification of 29 mesotheliomas cases, 26 lung cancer cases, 1 larynx cancer case, 3 ovarian cancer cases, and 7 asbestosis cases [5]. It is important to note that, as explained before, the focus of the Sibaté study has been primarily on mesothelioma because is a sentinel disease of asbestos exposure and there is a tight etiological link between them [5]. Additionally, focusing on mesothelioma enables the assessment of the presence of a cluster through a formal comparison of the figures observed in Sibaté with those in regions and areas where asbestos exposure has been confirmed. Comparisons for other ARDs are more challenging due to various factors, including multifactorial etiology for certain cancer sites.

### 4. Validation of the Cases

Validation of cases requires an analysis of the medical records, making it important to request the medical records as soon as a case is identified. It is crucial to emphasize that ethical protocols require explicit approval from patients or their family members for the collection of medical records. The initial request made to the ethics committee at Universidad de los Andes did not include the collection of medical records, and thus, an amendment was requested and subsequently approved. Once the amendment was granted, patients or their family members were contacted to obtain copies of the medical records.

In Colombia, each health center has its own requirements for providing copies of a patient’s medical record. In some cases, a letter signed by the patient or a family member sufficed, while in other instances, notarized letters and copies of the patient’s identification (i.e., *cédula de ciudadanía*) were requested. This process was complex and time-consuming, involving multiple individuals, and students from the Environmental Engineering program at Universidad de los Andes played a crucial role in facilitating it. Of the 29 self-reported mesothelioma cases identified through the questionnaires, a total of 17 medical records were obtained for this group, and the investigation proceeded with these 17 patients.

The validation process of these 17 self-reported cases followed the criteria proposed by the Italian Mesothelioma National Register, which classifies mesothelioma cases as *certain, probable, possible, to be defined*, and *not mesothelioma* based on the information available in the medical records [61]. A panel consisting of four pathologists (three from Colombia and one from Italy), a thoracic surgeon, and a radiologist reviewed the records individually [5]. The Colombian panel members reached a consensus on the classification of diagnosis for the 17 cases, while the pathologist in Italy conducted her own classification [5]. This independent classification process provided internal validation, and agreement among the assessments was generally high. There was only one case with a slight difference, as the pathologist in Italy classified it as either “not mesothelioma” or “to be defined”, while the Colombian panel classified it as “to be defined”. The consensus was to classify this case as “not mesothelioma” [5]. The final classification of the 17 mesothelioma diagnoses was 15 as “certain”, one as “probable”, and one as “not mesothelioma” [5].

### 5. Estimation of the Age-Adjusted Incidence Rate for Mesothelioma

Several decisions were made during this stage:

- To reduce recall bias, detailed characterization was limited to mesothelioma cases diagnosed during the previous decade (i.e., 2007–2017), totaling 13 cases [5]. Notably, the most concerning finding of this detailed analysis was the early age of diagnosis, suggesting that many cases might have been exposed to asbestos during childhood [5]. Among the 13 cases, one was diagnosed in their 30s, seven in their 40s, and five in their 50s [5].
- The estimation of the age-adjusted incidence rate was based solely on mesothelioma cases diagnosed while residing in Sibaté. This excluded cases that had previously lived in Sibaté but were diagnosed after relocating to other municipalities, resulting in a reduced the number of mesothelioma cases available for this estimation (9 cases, consisting of 6 males and 3 females) [5]. These estimates were computed using official population data for Sibaté, categorized by age and gender, obtained from the National Administrative Department of Statistics (Departamento Administrativo Nacional de Estadística—DANE).

To assess whether the incidence rate was elevated, it is necessary to have reference values for comparison. If this information is available for other regions in the country were the study is conducted, these reference values can be used. However, as previously mentioned, due to the issues with SISPRO, this option was not viable for the Sibaté study. Instead, the study relied on the data from the IARC report “Cancer Incidence in Five Continents Vol. X” [68].

The age-adjusted incidence rate in Sibaté for males was determined to be 3.1 per 100.000 persons-year, while for females, it was 1.6 per 100.000 persons-year [5]. In comparative terms, these rates are found to be notably high for both genders, with the rate for females being one of the highest in the world. This finding led to the conclusion that a mesothelioma cluster existed in Sibaté [5]. It is important to acknowledge that because of the not exhaustiveness of the applied structured interviews, these observed figures likely represent an underestimation of the actual number of cases in the population (i.e., conservative estimates).

### 6. Determining Potential Exposure Sources to Asbestos for the Cases

Three members of the research team independently analyzed the occupational history and other potential exposure sources to asbestos of the 13 *certain* mesothelioma cases, using the categories proposed by the Italian Mesothelioma National Register [61]. After reaching consensus, each case was classified into one or two categories [5]. The most important finding was that only one case had worked in the asbestos-cement facility [i.e., *certain professional exposure*) [5]. The remaining 12 *certain* mesothelioma cases had exposures classified in the categories *household exposure, non-professional exposure*, and *environmental exposure*. This revelation highlighted that asbestos exposure events had occurred in Sibaté outside the asbestos-cement facility. It is crucial to clarify that this finding does not rule out the possibility that a high number of current and former workers of the asbestos-cement facility have been diagnosed with mesothelioma or another ARD, and it could be the result of the low number of workers participating in the study. The reasons that lead to this low participation have not been determined.

### 7. Communicate with Health and Environmental Authorities and the Affected Resident Population the Study Findings and Disseminate Key Messages to the Public

Throughout the development of the Sibaté study, the communication of its findings served various purposes and occurred at different points in time. Before the publication of the first peer-reviewed article, the concerning finding of landfilled zones containing an underground layer of friable asbestos was reported to the municipality of Sibaté. This was communicated through several meetings with either the major or the heads of key secretaries. Later, when the municipality decided to replace pipelines in areas where the presence of the friable asbestos layer had been reported, meetings were convened with personnel from the Ministry of Health and Social Protection, the Ministry of Environment and Sustainable Development, the Governor’s office of the State of Cundinamarca (where Sibaté is located), and the municipality of Sibaté. A committee involving all the governmental institutions, Universidad de los Andes, and the French Research Institute for Development was established to monitor the situation. Additionally, members of the research team participated in a meeting at the State of Cundinamarca offices to provide further explanation.

The communication of the results was carried out according to a meticulously designed strategy, owing the gravity of the findings. The aim was to prevent panic among the residents of Sibaté and avoid stigmatization of the city and its inhabitants. The strategy encompassed drafting a press release in collaboration with the communication office of Universidad de los Andes, engaging with reporters to underscore the importance of delivering accurate messages, and conducting thorough review of the language used. Regarding the dissemination of the findings to the public, the results were conveyed through the mass media outlets, including newspapers, television, and radio news. This dissemination effort commenced in April 2019, following the validation by peers and the online publication of the first article of the Sibaté study.

## 4. Discussion

The operation of asbestos-processing facilities or asbestos mines poses an imminent health risk for the workers involved in these activities, their relatives, and for the residents who live in the vicinity [4,69,70,71,72]. Therefore, it is imperative to establish robust and reliable surveillance systems for both ARD and environmental asbestos contamination. Such systems facilitate early ARD diagnosis and the control and remediation of active asbestos sources. Unfortunately, asbestos is still predominantly used in low- and middle-income countries, which may lack the institutional capacity and technical knowledge to effectively manage the associated risks. Consequently, it is of paramount importance for governments to implement environmental monitoring strategies to identify potential sources or circumstances of asbestos exposure, along with epidemiological surveillance of populations at risk due to their residence location, para-occupational exposure, and/or occupation. The Italian Mesothelioma National Registry is an example of a successful model for surveillance.

In the absence of robust surveillance strategies or reliable health information systems, this article presents a methodology for studying the potential health impacts of asbestos-processing facilities on individuals residing in the facilities’ influence areas. However, this strategy is resource and time-intensive and may not be suitable for large populations. Therefore, the proposed protocol should be considered a non-definitive approach to understanding and addressing health risks in regions where such facilities operate.

An additional approach for validating self-reported ARD cases, currently in progress within the Sibaté study, though not included in this protocol, involves re-analyzing tissue samples preserved in paraffin blocks used for the initial medical diagnosis of ARD cases. Collecting tissue samples can be complex and may not always be feasible, and it also requires ethical approval. When attempted within the framework of the Sibaté study, it was revealed that each medical center had its own protocols and requirements for providing the tissue samples. Additionally, such analysis requires the involvement of a pathologist and incurs additional laboratory costs.

One of the primary limitations of the proposed strategy is the constraint on the number of individuals that can be followed and characterized, considering the potentially vast number of people at risk. A study conducted by members of our research group quantified this challenge in Colombia [4]. For instance, in Sibaté alone, based on the 2018 census, the population living near the asbestos-cement facility ranged from 12 individuals within a 500-meter radius to 932,838 individuals within a 10,000-meter radius [4]. Among those within the 10,000-meter radius, 304,842 people were aged 19 or younger [4]. Nationwide, in Colombia, where six asbestos-processing facilities and an asbestos mine used asbestos until the asbestos ban took effect on January 1st 2021, the 2018 census indicated 13,285 individuals lived within a 500-meter radius of these facilities and the mine, expanding to 6,724,677 individuals living within a 10,000-meter radius [4]. Given these figures, it is important to emphasize that the methodology proposed in this article does not replace the need for surveillance programs and reliable health databases. Such programs should be defined ad hoc in each country or area contemplating the priorities and the balance between costs and benefits, considering the economic costs, technical feasibility, the estimated time needed for their implementation, and the expected benefits in mitigating the health risk over time, among others.

The relatively low number of certain mesothelioma cases identified in Sibaté to date warrants further discussion. Colombia lacks comprehensive information on the history of the asbestos industry, leading to a lack of data on the number and characteristics of workers employed in the asbestos-cement facility in Sibaté. Additionally, active participation from the workers’ union has not been achieved, further limiting the ability to identify workers who may have developed ARD.

While the Sibaté study has primarily focused on mesothelioma thus far, it is essential to recognize the significance of all ARD, each of which has substantial and adverse societal impacts. In the future, the Sibaté study aims to encompass a broader spectrum of ARD.

Despite the concerning findings of the Sibaté study, as of September 2023, neither risk communication nor risk management plans have been implemented for Sibaté.

## 5. Conclusions

Implementing the protocol described in this article requires collaboration among experts from various disciplines, including epidemiologists, exposure assessors, physicians, engineers, geographers, social scientists (such as anthropologists, sociologists, psychosocial and communication experts), and public health experts, among others. The specific disciplines needed for the study may vary depending on the local health, environmental, social, and economic conditions of the area where the study is conducted. Expertise in asbestos-related issues is also indispensable. In the case of Sibaté, international collaboration and the willingness of experts from Italy and France to contribute to understanding the problem were pivotal to the study’s success. Such collaborations are highly recommended when addressing the complexity of public health problems arising from asbestos use.
